# Exploring the mechanisms of Qingdu Fang for the treatment of cervical HR-HPV using UPLC-QTOF-MS, network pharmacology, and cell experimentation 

**DOI:** 10.3389/fphar.2024.1415422

**Published:** 2024-07-10

**Authors:** Shanyun Wang, Guangru Li, Zhuqiang Wang, Qing Luo, Jianfeng Zeng, Jing Xiao

**Affiliations:** ^1^ The Second Clinical School of Guangzhou University of Chinese Medicine, Guangzhou, Guangdong, China; ^2^ Zhongshan Hospital of Traditional Chinese Medicine, Zhongshan, Guangdong, China; ^3^ Zhanjiang Institute of Clinical Medicine, Central People’s Hospital of Zhanjiang, Zhanjiang, Guangdong, China; ^4^ Zhanjiang Key Laboratory of Leukemia Pathogenesis and Targeted Therapy Research, Zhanjiang, Guangdong, China; ^5^ The Second Affiliated Hospital of Guangzhou University of Chinese Medicine, Guangzhou, Guangdong, China

**Keywords:** Qingdu Fang, HR-HPV, UPLC-QTOF-MS, Network pharmacology, cell experimentation

## Abstract

**Background:** Qingdu Fang (QDF) is a traditional Chinese herbal formula with remarkable clinical effect in the treatment of HR-HPV, but its mechanism remains unclear. In this study, UPLC-QTOF-MS was used to detect its components, network pharmacology was used to explore the traditional Chinese medicine monomers and their related targets for the treatment of HR-HPV in QDF. Molecular docking and *in vitro* experiments were performed to verify the results.

**Methods:** QDF constituents and active compounds were identified using UPLC-QTOF-MS analysis. TCMSP and GeneCard databases were used to identify active components, targets, and potential therapeutic targets in HR-HPV. PPI network was constructed using the String database to analyze protein-protein interactions. Cytoscape3.7.2 was used to construct PPI networks, while GO enrichment and KEGG pathway analyses with R. The effect of QDF on H8 cell proliferation was measured using the CCK-8 method, and apoptosis and cell cycle was assessed with flow cytometry. The effects of QDF on PI3K/AKT pathway were detected by Western blotting.

**Results:** A total of 27 compounds were identified on QDF by UPLC-QTOF-MS. Base on Network pharmacology,a total of 254 target genes are involved in the action of QDF on cervical HR-HPV. PPI analysis suggested that TP53, JUN, AKT1, STAT3, TNF and IL6 were potential targets for QDF treatment of HR-HPV. Molecular docking shows that two compounds have strong binding activity with AKT1. CCK-8 and morphological observation have shown that QDF inhibits H8 cell proliferation in a dose-dependent manner. Flow cytometry experiments suggest that QDF induces apoptosis and cell cycle arrest in H8 cells. Western blotting experiments reveal that QDF inhibits the PI3K/AKT signaling pathway.

**Conclusion:** QDF has a multi-faceted therapeutic approach for HR-HPV, targeting inflammation, oxidation, and apoptosis. It induces apoptosis in H8 cells by inhibiting the PI3K/AKT pathway.

## 1 Introduction

Cervical cancer is a significant global health issue, ranking fourth in incidence and mortality among female cancers worldwide. In China, there are 98,900 new cases and 30,500 deaths annually, with numbers continuing to increase (Chang et al., 2022). Numerous studies have shown that long-term HR-HPV infection is the main cause of cervical cancer (Bowden et al., 2023). HR-HPV infection can cause cervical cancer over a 10–15 year period (Meenu et al., 2023). Currently, cervical cancer treatment primarily involves surgery, radiotherapy, chemotherapy, targeted therapy, and immunotherapy (Hill, 2020). However, there are shortcomings in late-stage treatment, including low effectiveness and recurrence. Early intervention can prevent cervical cancer by eliminating HPV infection before it becomes irreversible. Currently, there is no standard treatment for cervical HR-HPV infection. The lack of availability of HPV vaccines in developing countries hinders widespread use (Ojone and Adeola, 2023). Waiting for the body to clear the virus naturally may be cost-effective, but could also cause mental stress and delay treatment. Studies have found that interferon therapy has a low short-term success rate for persistent HR-HPV infection in the cervix, with less than 50% effectiveness and minimal side effects (Li and Xiong, 2022). Other physical therapies like cryotherapy, laser, and surgery also cannot completely eliminate HPV infection (Fu and Jin, 2023). Clinical observation and laboratory studies also show that TCM has its advantages on treating the virus, and the research and development of new drugs or TCM treatments for infectious diseases has broad prospects (Chen et al., 2017; Lv, 2022).

QDF is a TCM prescription used for high-risk HPV infection (Liu, 2021), the HR-HPV negative rate was 73.91% after 7 months, but its exactly molecular mechanism is unclear. This study used various techniques to explore the potential targets and pharmacological mechanism of QDF. The study aimed to identify therapy targets for HR-HPV, construct a network of QDF target HR-HPV interactions, and explore the molecular mechanism and signaling pathway involved in treating HR-HPV. The PI3K/AKT signaling pathway is important in promoting tumor proliferation by inhibiting apoptosis and stimulating the cell cycle (Zhang et al., 2021). This study aimed to explore how QDF inhibits cell growth and induces cell appotosis by affecting the PI3K/AKT signaling pathway.

## 2 Materials and methods

### 2.1 Confirm the constituents of QDF through liquid chromatography-mass spectroscopy (UPLC-QTOF-MS)

The components of QDF were analyzed by Ultra-high Performance Liquid Chromatography-Quadrupole Time-Of-Flight Mass Spectrometry. The chromatography was performed on Zorbax eclipse Plus C18 column (2.1 mm × 100mm, 1.8 μm). The QDF lyophilized powder obtained in section 2.3.3 was dissolved with 20% acrylicitrile solution and filtered with 0.22 μm membrane filter. The sample size was 1.0 µL.The following conditions are used:The mobile phase is acidified water (0.1% formic acid, A) and acetonitrile(B); The column temperature was set at 40°C, and the flow rate was set at 0.3 mL/min, including the following sequence: 0–1min: 10%B; 8–16 min:40%–55%; 16–40 min:90% B; 40–43 min:10%; Mass Spectrometry parameters were set as follows: drying temperature was 320°C, drying gas flow rate was 8L/min, sheathing temperature was 350°C, sheathing gas flow rate was 11L/min, atomizing gas pressure was 35psi, and fragmentation voltage was 135 V. Capillary voltage is 3500 V (+)/3500 V (−), Nozzle voltage is 1,000(+)/1,000(−), real-time calibration reference ion is 121.0508/922.0098 (+), 112.9856/1,033.9881 (−); The full scan collection parameters are set as follows: Quality range: 100–3,200 m/z, Acquisition rate: 1 MS/s; The automatic MS/MS acquisition parameters are as follows: MS mass range is 100–3000m/z, MS acquisition rate is 4 MS/s, MS/MS mass range is 100–3000m/z, MS/MS acquisition rate is 2 MS/s, collision energy is 10, 20, 40eV, 2 parent ions/cycle, MS/MS mass range is 2 MS/s.

Parent ion threshold is 200 counts, 0.01%, dynamic exclusion is 1 mass spectrometry after exclusion, release time is 0.08min, quadrupole resolution is medium (4 Da). Finally, Agilent TCM database was used for data analysis.

### 2.2 Network pharmacology

#### 2.2.1 Collection of QDF targets

The target genes of the active components of QDF were identified using Traditional.

Chinese Medicine Systems Pharmacology database (TCMSP, https://old.tcmsp-e.com/tcmsp.php). Compounds with oral bioavailability (OB) ≥ 0.3 and drug-like index (DL) ≥ 0.18 were considered. Therefore, QDF has potential activities, with its active components cross-referenced with the TCMSP database and gene naming standardized using Gene Cards. The Uniprot databases were used to collect disease targets, which were then combined and any duplicates were removed to obtain HR-HPV disease-related targets.

#### 2.2.2 Acquisition and collection of disease targets

The keyword “HR-HPV” was searched in the GeneCards database (https://www.Genecards.org) to indentify HR-HPV-related targets. The intersection of QDF targets with HR -HPV targets was performed using veny2.1.0 (https://bioinfogp.cnb.csic.es/tools/venny/).

#### 2.2.3 Construction and analysis of the protein-protein interaction (PPI) network

The above intersection targets were imported into the String database (https://cn.string-db.org/)with a confidence score threshold of >0.9, and abnormal targets are excluded. Analysis was limited to “*Homo sapiens*” species for interaction relationships. The PPI network TSV file was visualized in Cytoscape 3.7.2, identifying core targets with a screening count of ≥3 times.

#### 2.2.4 Biological pathway analysis

The common targets data was imported into the DAVID database (https://david.ncifcrf.gov/) for gene list analysis in *H. sapiens*. Functional annotation was done using GO enrichment and KEGG pathway analysis with screening qualifiers of *p* < 0.05 and FDR<0.05 to identify biologically significant processes related to diseases.

#### 2.2.5 Targets-pathway network analysis

The top 20 KEGG pathways were chosen based on the top 25 targets identified through protein-protein interaction analysis. A network diagram was created to illustrate the relationship between targets and pathways.

#### 2.2.6 Key active compounds and the targets verify

To assess the connection between the target and compound, we used molecular docking with compounds containing the most relevant critical genes and shared core genes. Protein structures were obtained from the PDB database (https://www.rcsb.org/) and the two-dimensional structure of the small molecule ligand of the active ingredient was obtained from the PubChem database (https://pubchem.ncbi. nlm.nih.gov). CB-DOCK was used to predict protein-compound binding activities. Credibility perceptions were measured by assessing docking affinity, with a value below −7.0 kcal/mol indicating a strong bond. The Ligplot software (Version 2.2) was used to show the optimal dock group.

### 2.3 Cellular experiments

#### 2.3.1 Cell line and reagents

The HPV16 immortalized human cervical epithelial H8 cell line was kindly donated by Department of Gynecology, Guangdong Hospital of traditional Chinese medicine, furnished by the Biophysics Department, Institute of Basic Medicine, Chinese Academy of Medical Sciences.

CCK-8 Kit (Meilunbio,MA0218), Annexin V-FITC/PI Apoptosis detection Kit (Meilunbio, MA0220), fetal bovine serum (Biological Industries, 04-001-1ACS); BCA Protein Assay Kit (BeyotimeBiotechnology,P0010S); anti-TLR4; (Santa,sc-293072),anti-PI3K(Affinity,AF6241); anti-AKT (Affinity,AF6264),anti-phospho-AKT (Affinity,AF0016); Goat Anti-Rabbit IgG (H+L) HRP (Affinity,S0001).

#### 2.3.2 Cell culture

H8 cells were cultured in a medium with 10% fetal bovine serum in an incubator with 5% CO2 at 37°C. The cells were passaged when the fusion reached 80%–90%.

#### 2.3.3 Formulation of QDF

The herbs used in the study came from Zhongshan Hospital of Traditional Chinese Medicine and were verified by researcher Peng Weiwen. The QDF contained Huang Bo (10 g), Gan Cao (10 g), Tubeimu (10 g), and Daqingye (15 g).

The herbs were soaked in 1,000 mL of water for 30 min, then decocted twice for 90 min each. The resulting solution was filtered through two layers of gauze and concentrated using rotary evaporation at 60°C. The concentrated solution was frozen at −80°C overnight, and the powder with a yield of 6.38g/g^-1^ was obtained after vacuum freeze-drying. The weight of the lyophilized powder was calculated by dividing the weight of the physical liquor by the weight of the original herbs. Dissolve 2 g freeze-dried powder in 40 mL PBS at 80°C for 30 min. The solution was centrifuged at 1,000 r/min for three cycles of 10 min each, and the supernatant was filtered using a 0.22 μm filter membrane. The lyophilized powder was concentrated at 50 μg/mL in the physical liquor and stored at −20°C for long-term preservation before extraction at the predetermined location.

#### 2.3.4 CCK-8 experiment

A cell suspension with a final density of 5 × 10^4^cells/mL was seeded into each well of a 96-well plate with 100 µL of the suspension containing 5,000 cells per well. The cells were then incubated for 24 h. Blank controls using only complete medium were also set up. The cells were treated with different concentrations of QDF for 24 h. After removing the original medium, a culture solution containing CCK-8 reagent was added to each well and incubated at 37°C for 1–4 h. Absorbance values were measured using a MULTISKAN FC(Thermo, United States) with two replicates per group and three replications. The reader measured absorbance at 450 nm. Cell viability was calculated using a formula and GraphPad Prism 8.0.0 software was used for data analysis.

#### 2.3.5 Cell morphology observation

Cells were initially plated at a density of 1×10^6^ cells/well in 6-well plates with 2 mL of media. After 24 h of incubation at 37°C and 5% CO2, cells were treated with four concentrations of QDF (0 g/L, 1 g/L, 2 g/L, and 3 g/L) for 24 h. Morphological changes were observed using a phase contrast inverted microscope.

#### 2.3.6 Apoptosis assessed by flow cytometry analysis

Cells were treated according to the outlined procedure in section “2.3.5,” then incubated in 6-well plates for 24 h. After harvesting, the cells were washed twice with PBS. A total of 1–5×10^5^ cells were harvested and treated with 5 μL of Annexin V and PI reagents. The samples were then incubated at room temperature for 10 min, followed by the addition of 10 μL of PI and further incubation in the dark for 15 min. The process was done and analyzed using a CytoFLEX (Beckman, United States) and CytoExpert software within 1 h, repeated 3 times.

#### 2.3.7 Evaluation of influence on cell cycle

Cells were treated as outlined in section “2.3.5,” incubated in 6-well plates for 24 h, harvested, washed with PBS twice, and then incubated with PI/RNase Staining Buffer (Thermo, United States) for 10 min at room temperature in the dark. After mixing with PI/RNase Staining Buffer, the samples were incubated in darkness for 15 min. They were then analyzed using a CytoFLEX within 1 h. The experiment was repeated three times.

#### 2.3.8 Western blot analysis

H8 cells were cultured in 100 mm dishes and divided into two groups: administration and blank. The administration group received QDF at concentrations of 1 g/L, 2 g/L, and 3 g/L. After 24 h, the supernatant was removed, cells were collected, washed with PBS, and the liquid was discarded. A protein lysis solution was made with RIPA buffer (Beyotime Biotechnology, P0013B) and PMSF(Beyotime Biotechnology,ST506) at a 99:1 ratio. Cells were lysed at 4°C for 30 min, then centrifuged at 150,00× *g* for 10 min at 4°C. The supernatant containing all the protein was collected. Protein concentration was determined using a BCA Protein Assay Kit. After separating the sample proteins using SDS-PAGE gel electrophoresis, they were transferred to a PVDF membrane. The membrane was then blocked with a qick block for 15 min at room temperature before being incubated with the primary antibody overnight at 4°C.The membranes were washed, incubated with secondary antibodies, washed again, and then exposed to an ECL solution before capturing the protein bands with a chemiluminescence imaging system.

#### 2.3.9 Statistical method

Statistical analysis was conducted using SPSS26, Graphpad Prism 8.0.0, ImageJ, CytoExpert, and Flowjo software. The data were presented as mean ± standard deviation (SD), and group comparisons were performed using the Student’s t-test.

## 3 Results

### 3.1 Confirm the constituents of QDF through UPLC-QTOF-MS

**FIGURE 1:**
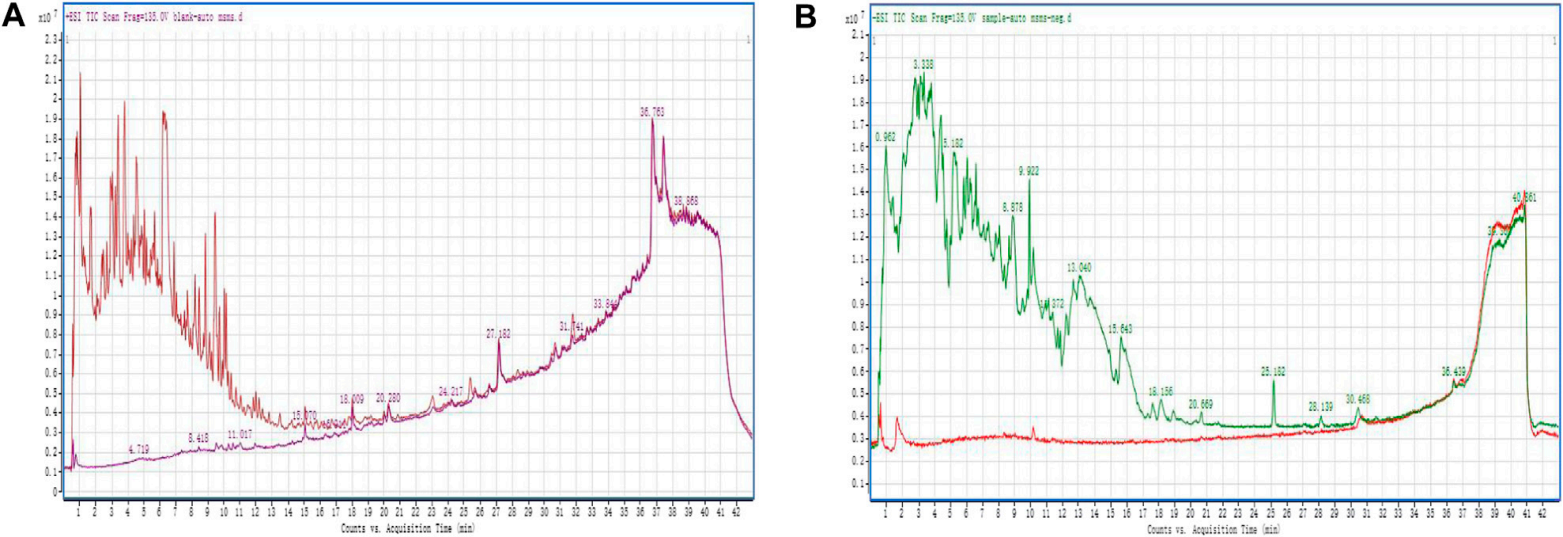
Positive and negative ion scan modes of QDF **(A)** positive ionization modes; **(B)** negative ionization modes.

QDF was analyzed using UPLC-QTOF-MS to identify its main components, including Glycyrrhiza uralensis Fisch and Phellodendron chinense Schnei, in both positive and negative ionization modes. The study observed reference ions at m/z823.4109, m/z342.174, m/z336.1231, and m/z821.3971, m/z336.1277, m/z342.174 with corresponding retention times. Indigowoad Leaf and Rhizoma Bolbostematis compounds were detected using negative ionization mode with specific retention times (Figure 1). Berberine, diammonium glycyrrhizinate, and phellodendrine were identified in both positive and negative ionization modes. Indirubin and tubeimoside A were only detected in negative ionizationmode.

### 3.2 Network pharmacology

#### 3.2.1 Active ingredients in QDF

QDF components were selected based on criteria from TCMSP databases, including OD and DB. The final list included 141 active ingredients, including 34 from Huangbai, 11 from Tubeimu, 7 from Daqingye, and 89 from Gancao. Among them, the common component of Huangbai and Gancao is quercetin, the common component of Daqingye and Gancao is Glycyrol, the common component of Huangbai and Daqingye is poriferast-5-en-3beta-ol, and the common component of Fritillaria and licorice is sitosterol. The common component of Huangbai, Tubeimu and Daqingye is beta-sitosterol. In addition, 254 QDF targets were obtained from the TCMSP database.

#### 3.2.2 Construction of active component of QDF-target network diagram

**FIGURE 2:**
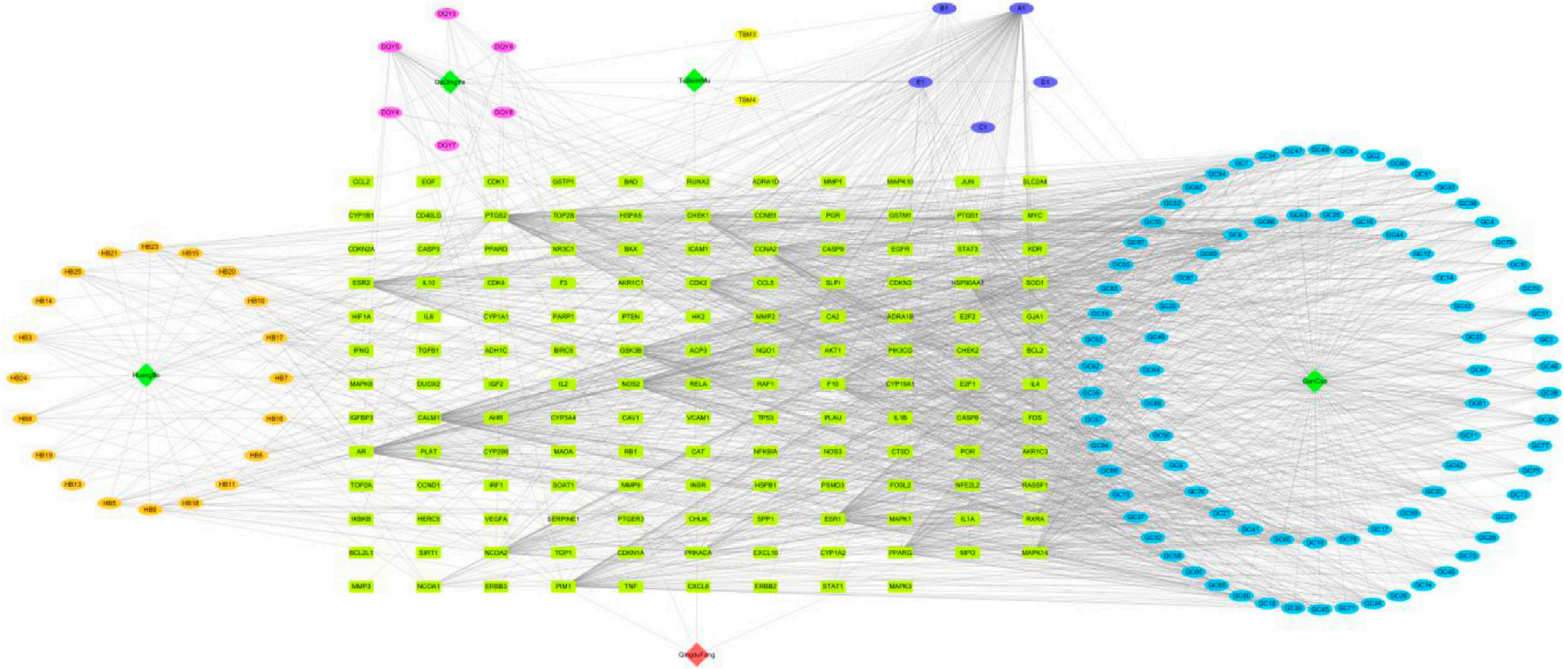
Active component of QDF-target network was constructed.

Cytoscape3.7.2 software was used to visualize the active components and potential targets of QDF. The ingredients of QDF, including Huangbai, Tubeimu, Daqingye, and Gancao, were found to engage with 254 targets. This demonstrates the multi-component-multi-target effect of QDF in treating HR-HPV (Figure 2).

#### 3.2.3 Prediction of QDF targets for the treatment of cervical HR-HPV

**FIGURE 3:**
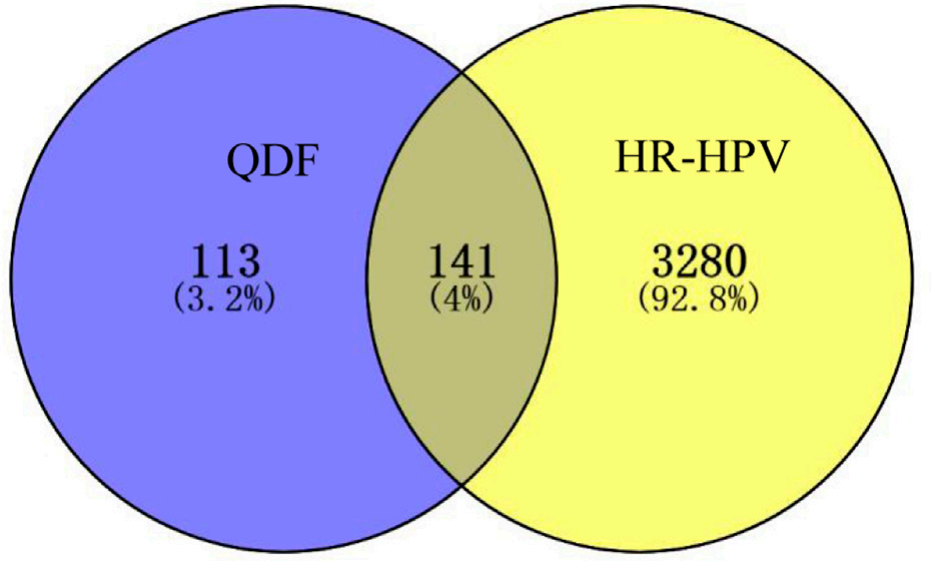
Venn diagram of QDF and HR-HPV targets intersection.

A thorough analysis identified 254 constituents QDF-related targets and 3415 HR-HPV-related targets. The intersection of active components and disease targets is showed by a Venn diagram and 141 commom targets were found (Figure 3).

#### 3.2.4 PPI network of QDF targets for the treatment of HR-HPV

**FIGURE 4:**
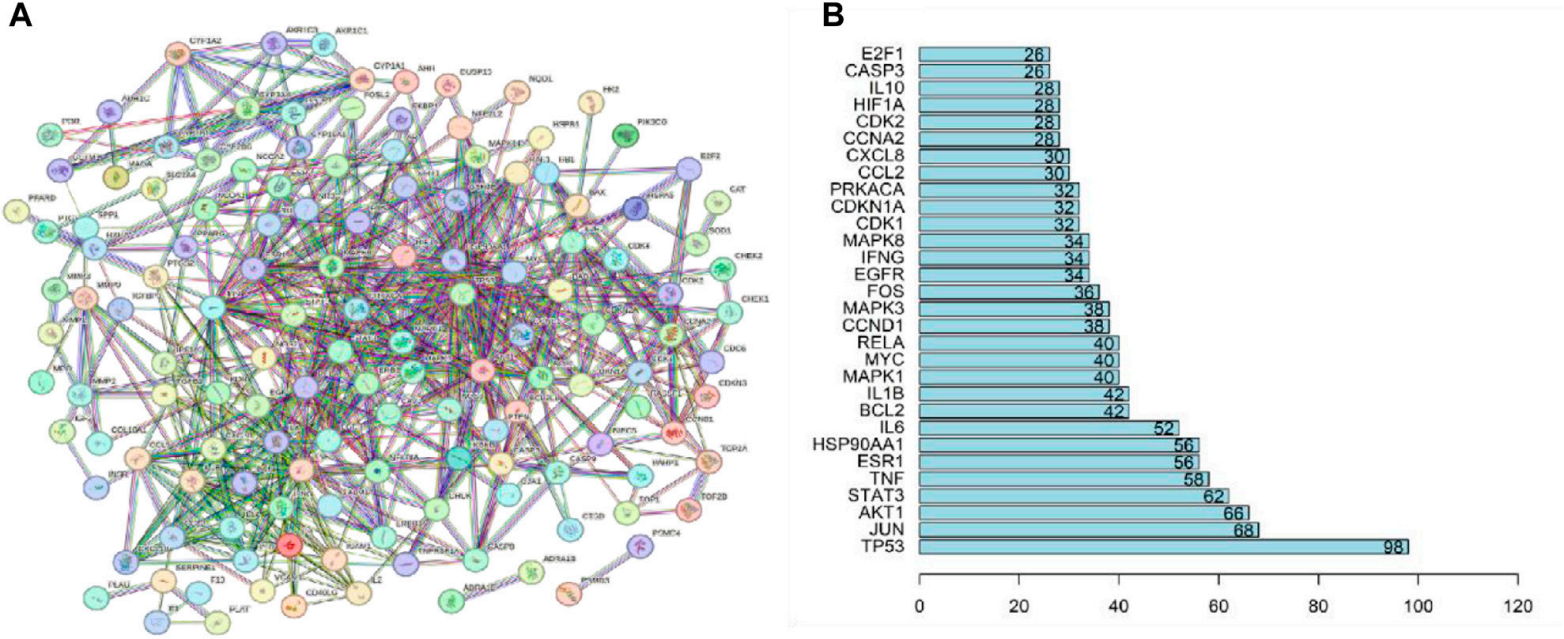
Protein-protein interaction network of common targets of QDF and HR-HPV. **(A)** PPI network of 254 key targets of QDF treating HR-HPV, **(B)** The top 20 targets with degree values are shown.

The common interaction targets of QDF and HR-HPV were input into the STRING database, screened with a confidence score threshold of >0.9, and the remaining parameters remained unchanged, and the PPI interaction network map was constructed (Figure 4A), resulting in a PPI network map with 682 protein relations involving 141 protein targets. Core targets were identified with a screening condition of count ≥3 times. The results indicate that TP53, JUN, AKT1, STAT3, TNF, ESR1, IL6, and HSP90AA1 could be potential targets for treating HR-HPV with QDF (Figure 4B).

#### 3.2.5 GO enrichment analysis and KEGG pathway analysis

**FIGURE 5:**
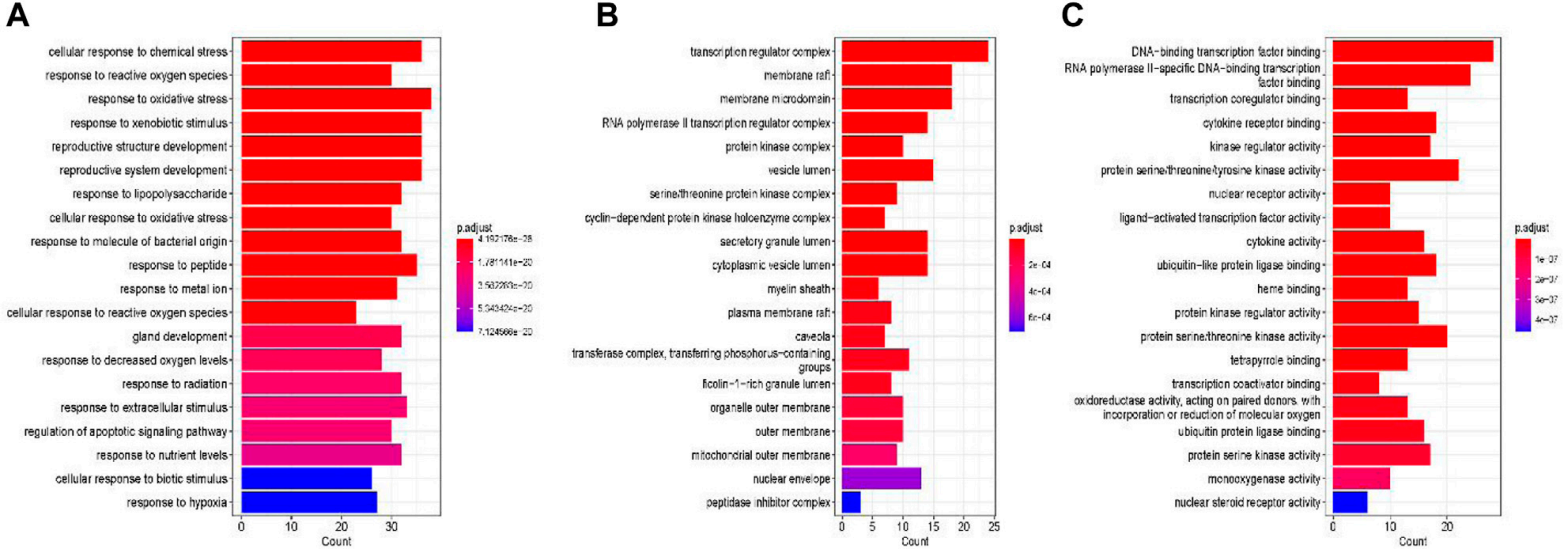
GO enrichment analysis. **(A)** biological processes, **(B)** cellular components **(C)** molecular functions.

Gene ontology (GO) enrichment analysis is a gene expression differential analysis method based on the GO functional classification system, which can be used to explore the biological processes (BP), cellular components (CC) and molecular functions (MF)of genes. GO enrichment analysis was performed using the DAVID database.254 gene functional analyses were conducted, resulting in 2,539 entries in the GO enrichment analysis, of these, 2,338 (*p* < 0.05)are related to BP (e.g.,.cellular response to chemical stress, response to reactive oxygen species, response to oxidative stress, response to xenobiotic stimulus), 39 are related to CC (e.g.,.transcription regulator complex, membrane raft, membrane microdomain, RNA polymerase II transcription regulator complex), and 162 are related to MF (e.g.,.DNA-binding transcription factor binding, RNA polymerase II-specific DNA-binding transcription factor binding, transcription coregulator binding, cytokine receptor binding) (Figure 5).

**FIGURE 6:**
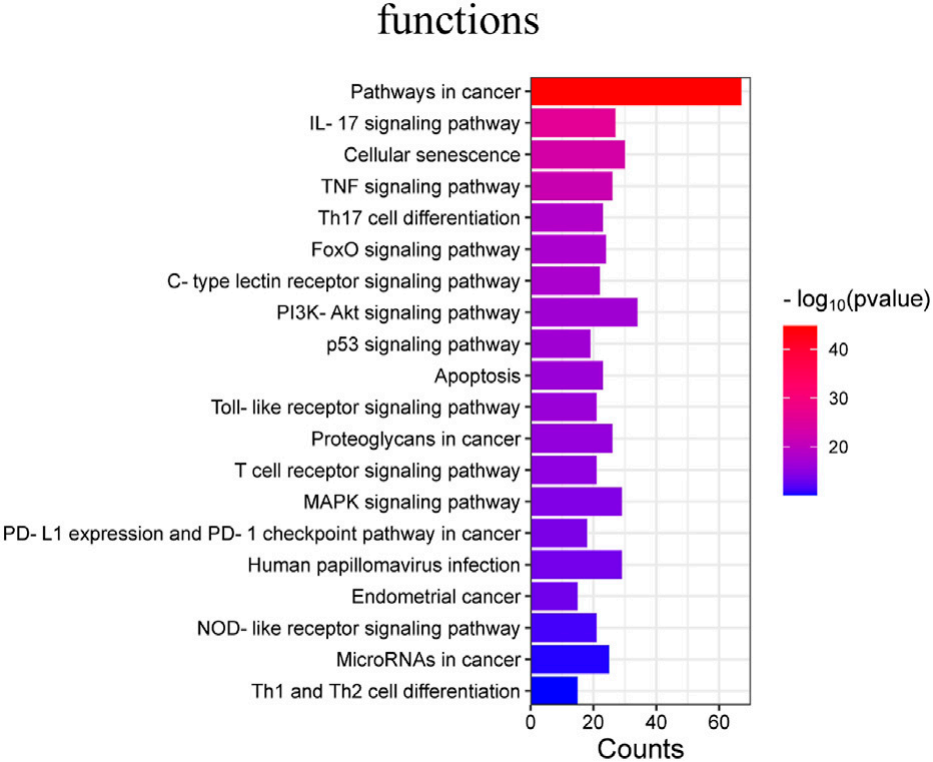
KEGG pathway analysis.

In addition, 177 KEGG pathways were obtained after KEGG pathway analysis (*p* < 0.05). The main signaling pathway are:Lipid and atherosclerosis, Pancreatic cancer, Prostate cancer,AGE-RAGE signaling pathway in diabetic complications, Hepatitis B (Figure 6).

#### 3.2.6 Construction of target-pathway network

**FIGURE 7:**
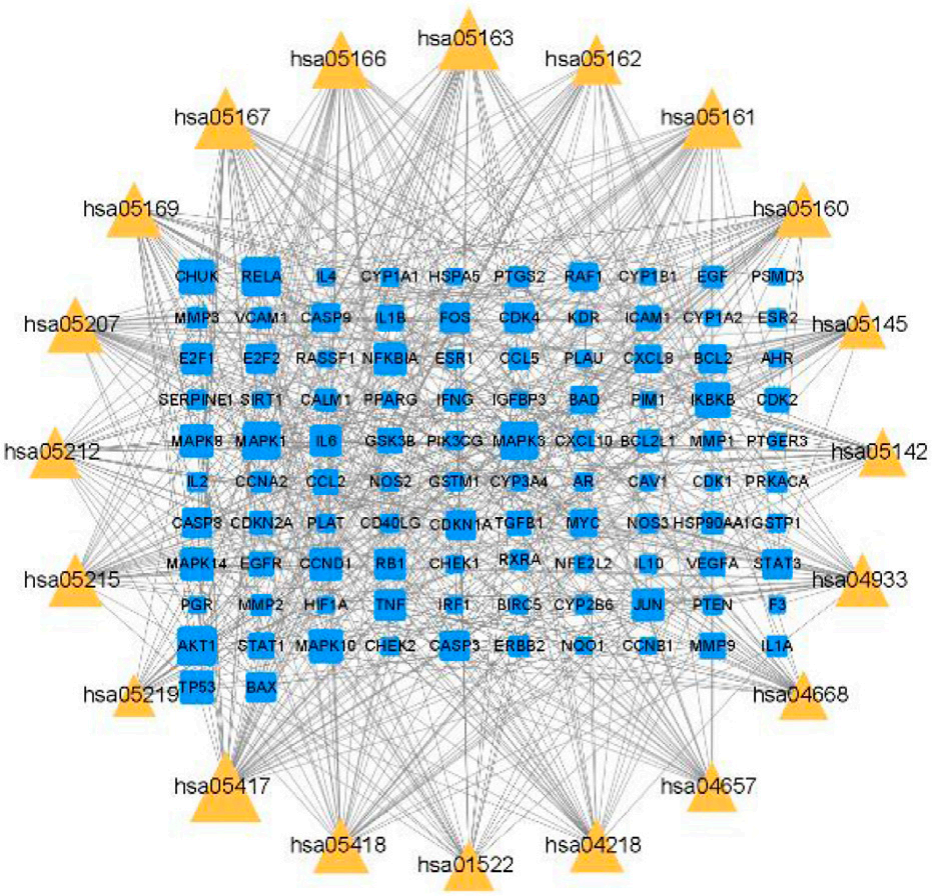
Target-pathway network analysis.

The top 20 targets of the PPI network were matched with the top 20 KEGG pathways to create a network map of target-pathway network. In Figure 7, QDF treats HR-HPV through multi-target and multi-pathway.

#### 3.2.7 Molecular docking verification of key targets

**FIGURE 8:**
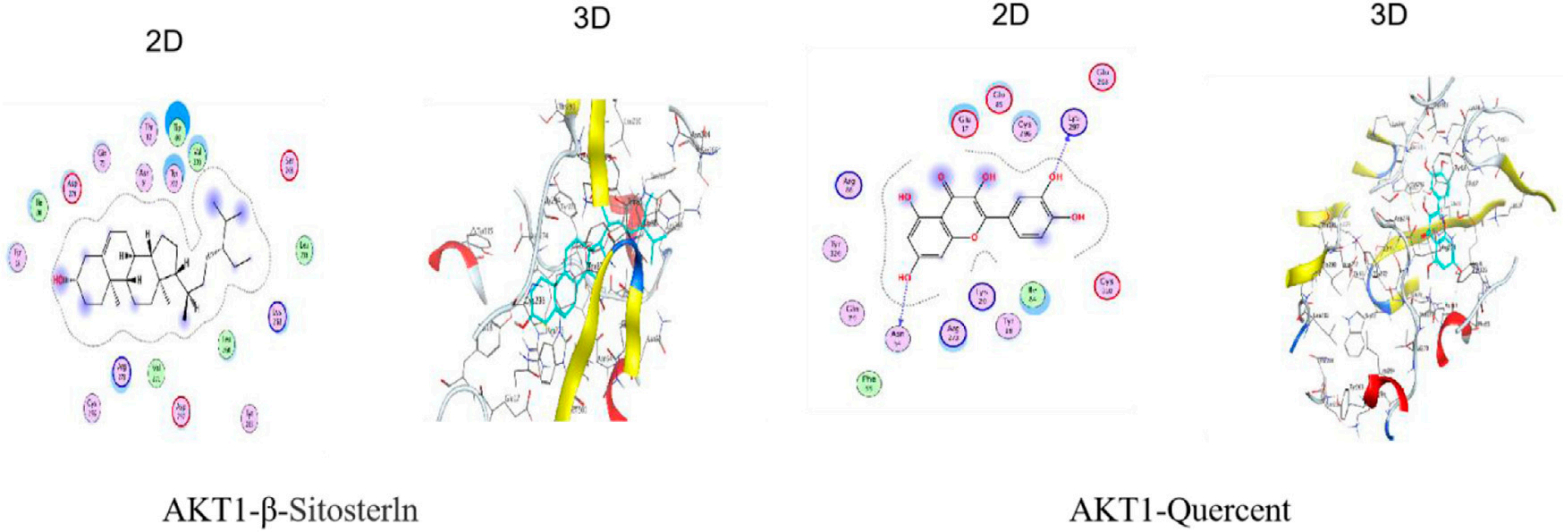
Molecular docking resluts of QDF with AKT1.

Molecular docking was done with AutoDock Vina to study how QDF interacts with the core target AKT1. Lower docking scores indicate stronger affinity between the ligand and receptor. The binding affinities of different target-compound pairs were calculated and shown in Table 1 and Figure 8.

**TABLE 1:** Results of molecular docking verification of key targets.

Name (PDBID)	Binding energy/(kcal·mol^−1^)	
	β-Sitosterln	Quercetin
AKT1(7NH5)	−8.6	−6.2

### 3.3 Experimental results

#### 3.3.1 Cytotoxicity of QDF *in vitro*

**FIGURE 9:**
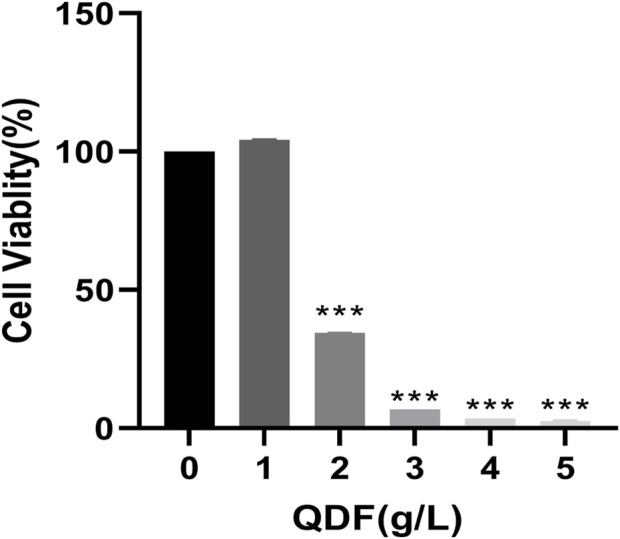
Cell activity of H8 after QDF treatment for 24 h.All data are presented as the mean ± SD, n = 3,*p < 0.05, **p < 0.01, ***p < 0.001.

To investigate the effect of QDF on H8 cells, different concentrations of QDF were treated and detected by CCK8.In Figure 9, the inhibitory effect on H8 cells increased with higher concentrations of QDF, showing a concentration-dependent relationship.

#### 3.3.2 Effect of the QDF on H8 cells

**FIGURE 10:**
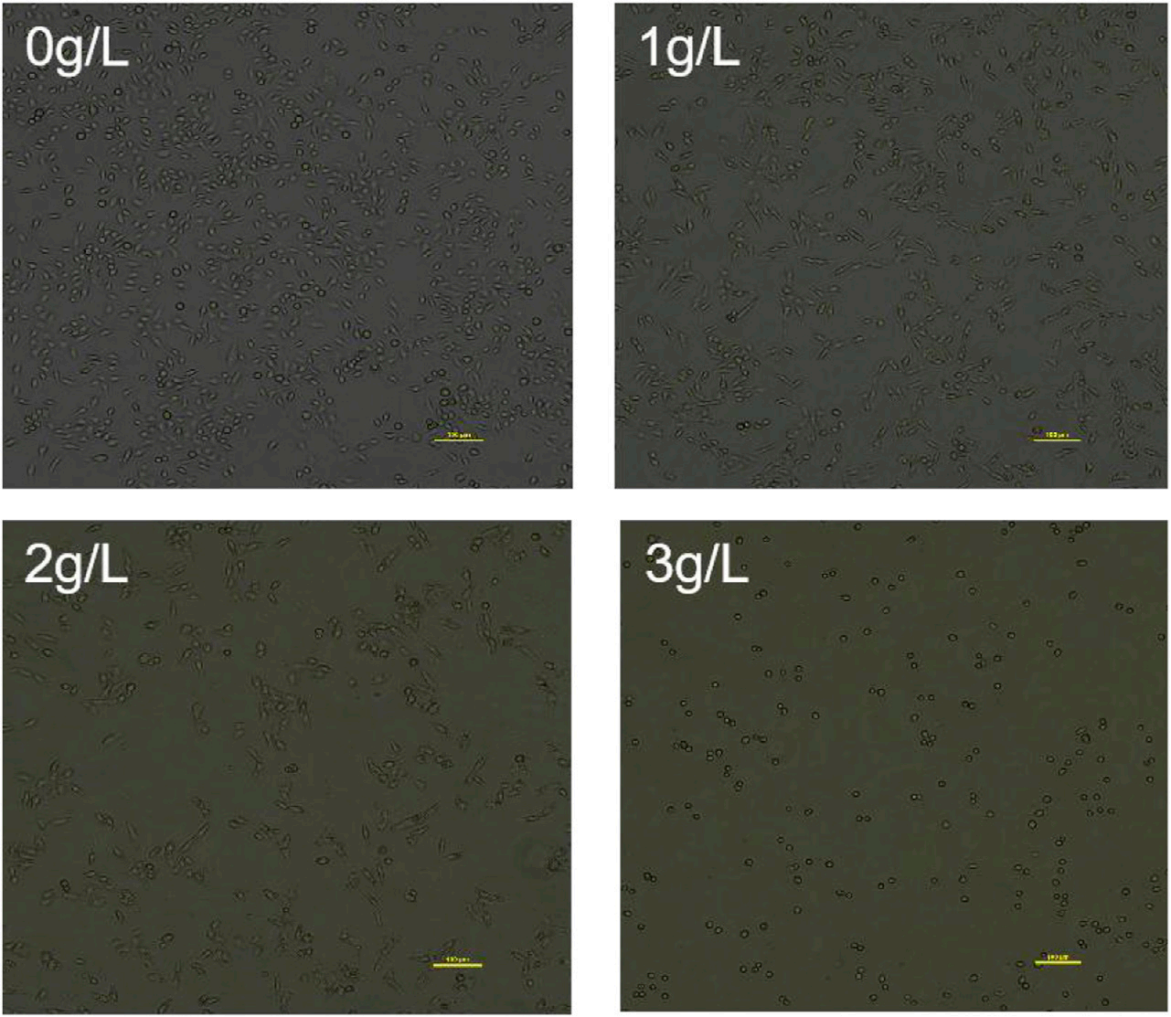
Effect of QDF on H8 cell morphology.

As shown in Figure 10, the cells in the control group grew well and were tightly attached to the wall. The cells grew spindle-shaped or polygonal, with full cell bodies and tight adhesion. With the increase of the concentration of QDF in the administration group, the cell density decreased, the cell size varied, the cell body gradually shrank and became round, and the cell shedding increased. QDF significantly inhibited the proliferation of H8 cells, and the results of CCK-8 were consistent.

#### 3.3.3 The effects of QDF on H8 cell apoptosis by flow cytometry

**FIGURE 11:**
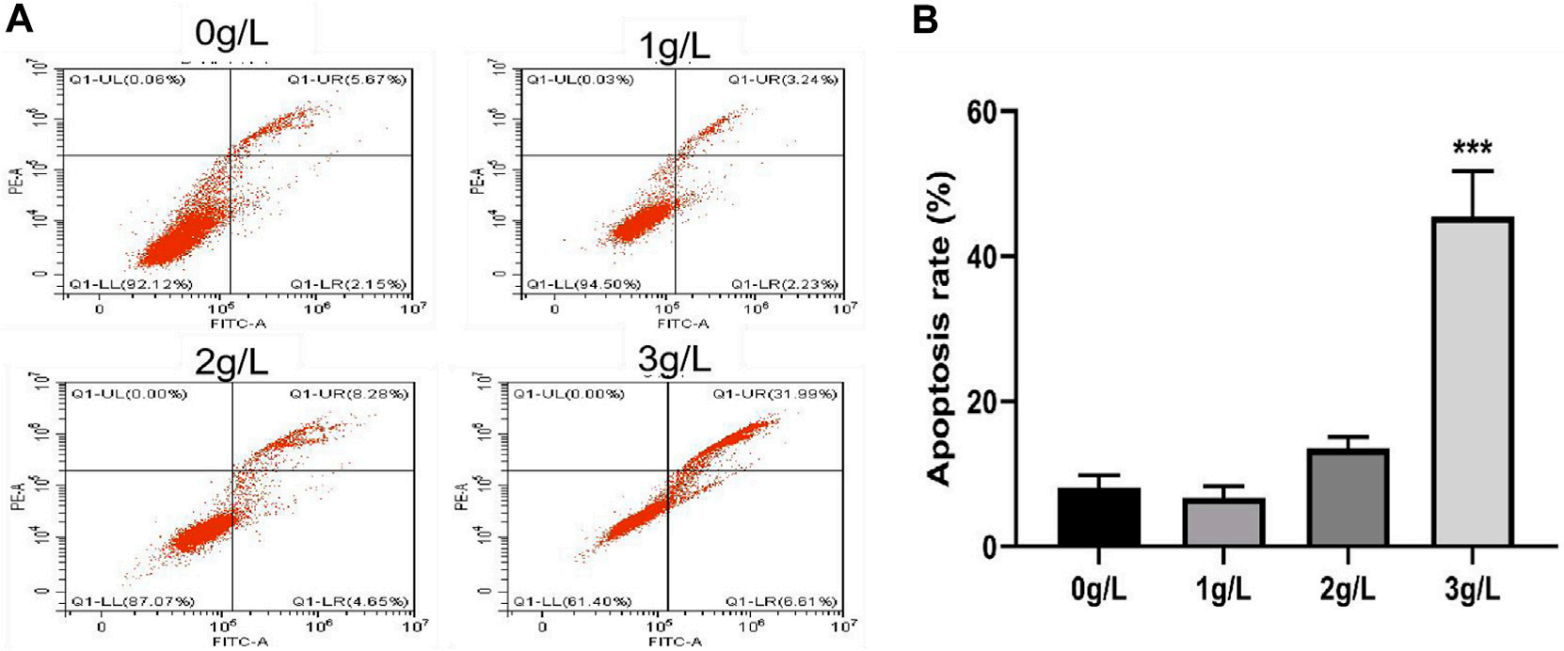
Experimental study on apoptosis of H8 cells induced by QDF *in vitro*. **(A)** Representative flow cytometry results of the effect of QDF on H8 cell apoptosis. **(B)** Statistical analysis results. All data are presented as the mean ± SD, n = 3. ***p < 0.001.

As shown in Figure 11, after treating H8 cells with different concentrations of QDF for 24h, the apoptosis effect on H8 cells gradually increased with the increase of the concentration of QDF, which had a concentration dependent effect, showing a concentration-dependent effect, suggesting a correlation between concentration and anti-apoptotic effects. The apoptosis rates in the blank control group and groups treated with 1 g/L, 2 g/L, and 3 g/L of QDF were (8.12 ± 1.39)%, (6.75 ± 1.23)%, (13.52 ± 1.32)%, and (45.49 ± 5.12)%, respectively. The apoptosis rate in the 3 g/L group was significantly higher than the blank control group (t = 32.780, *p* < 0.001) and the 1 g/L and 2 g/L groups (t = 7.412, *p* = 0.002).

#### 3.3.4 The effects of QDF on cell cycle of H8 cells by flow cytometry

**FIGURE 12:**
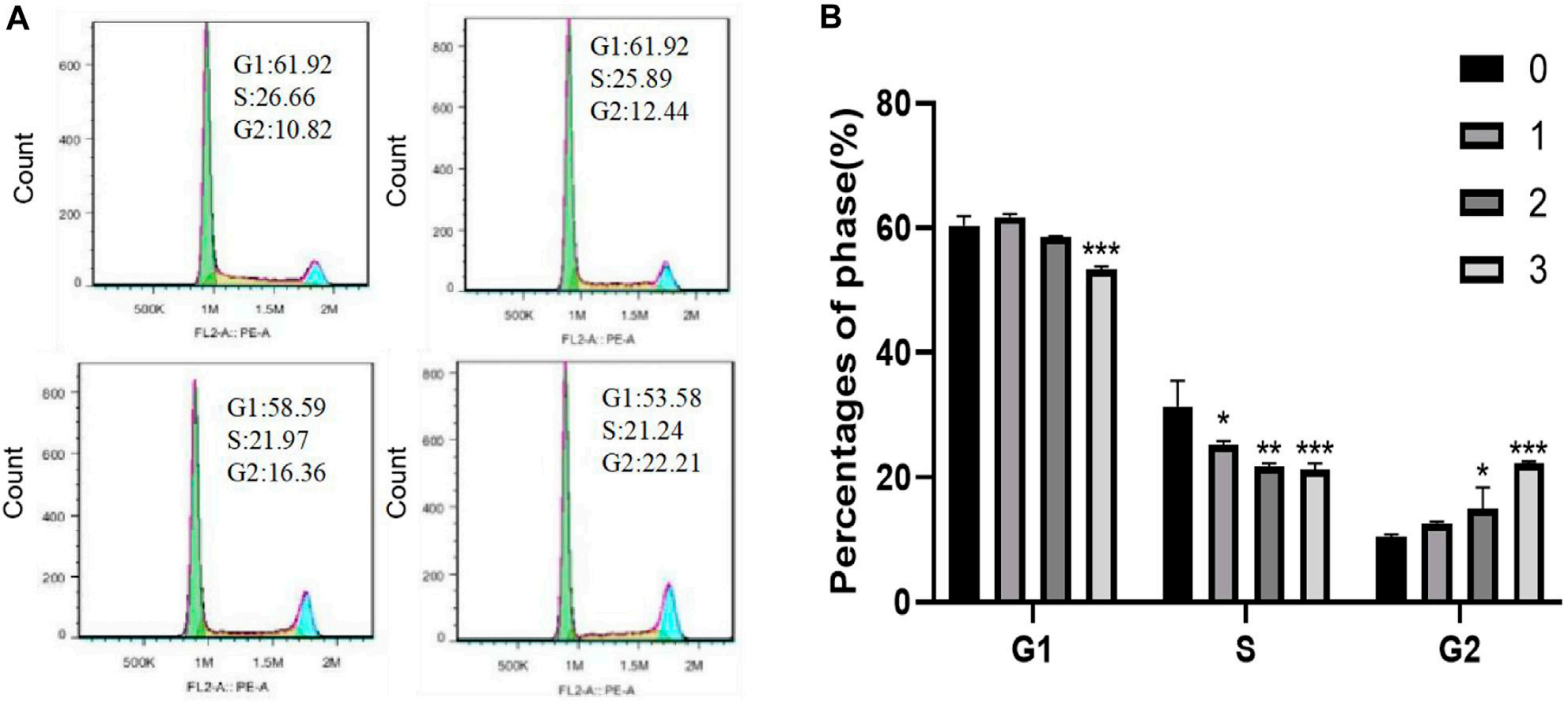
Experimental study on cell cycle of H8 cells induced by QDF *in vitro*. **(A)** Representative flow cytometry results of the effect of QDF on H8 cell cycle arrest. **(B)** Statistical analysis results. All data are presented as the mean ± SD, n = 3,*p < 0.05, **p < 0.01, ***p < 0.001.

Figure 12 shows that treating H8 cells with QDF for 24 h led to a significant increase in cells in the G2 phase and a decrease in cells in the G1 and S phases compared to the control group (*p* < 0.05).

#### 3.3.5 QDF inhibited the activation of the PI3K/AKT signaling pathway

**FIGURE 13:**
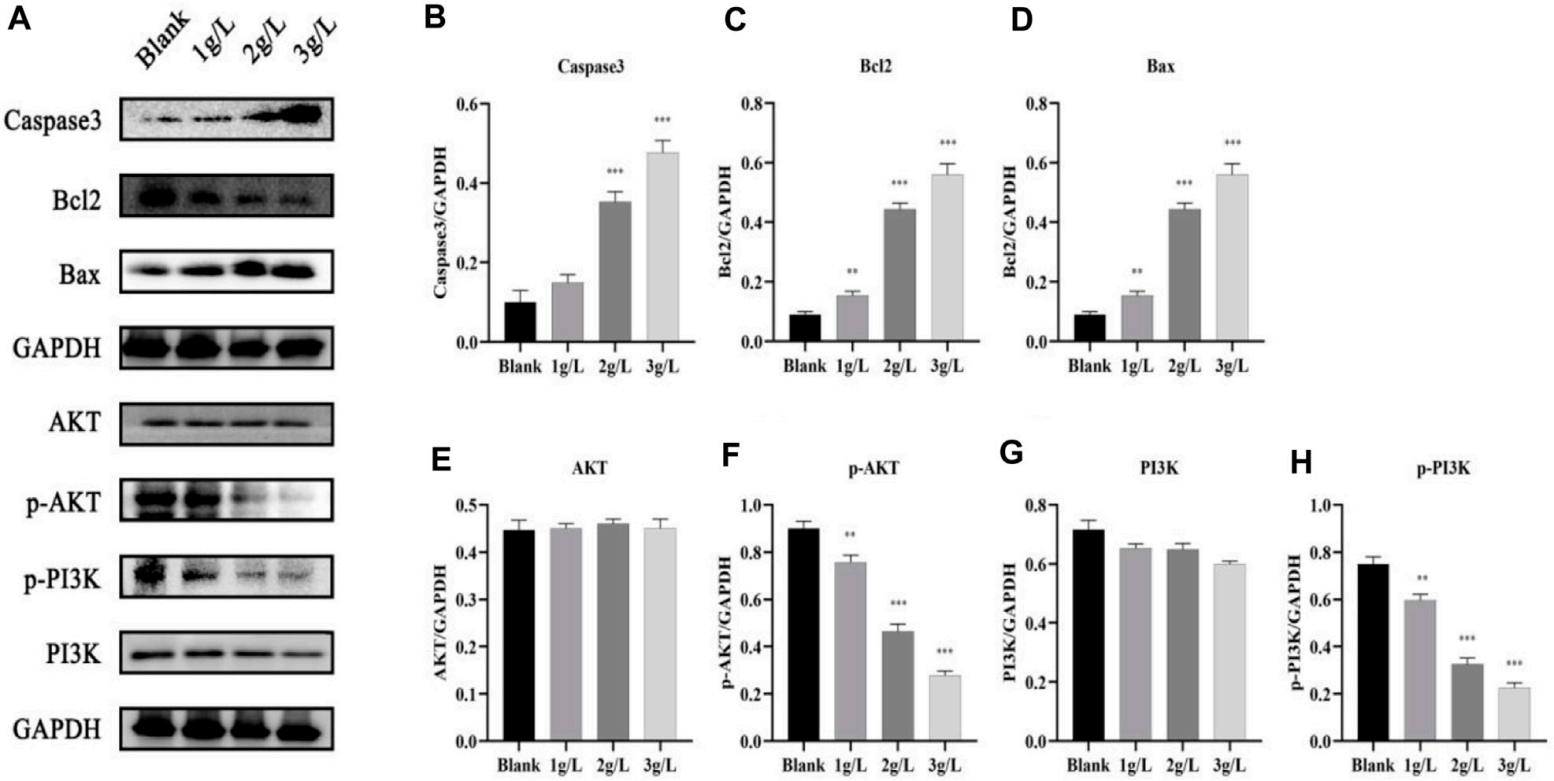
Expression of PI3K/AKT pathway protein. **(A)** Expression of PI3K, AKT, P-PI3K, p-Akt, Bcl2 and Bax proteins. **(B–H)** Relative protein expression of PI3K, AKT, P-PI3K, p-AKT, Bcl2 and Bax. All data are presented as the mean ± SD, n = 3,*p < 0.05, **p < 0.01, ***p < 0.001.

After treating H8 cells with different amounts of QDF for 24 h, Western blot analysis showed increased levels of caspase-3 and Bax, and decreased levels of BCL-2 in the PI3K/AKT pathway related proteins as QDF concentration increased. The study found that p-PI3K and p-AKT levels significantly decreased with QDF concentration, but there was no significant difference in PI3K and AKT levels at different concentrations of QDF (Figure 13).

## 4 Discussion

HPV, a common sexually transmitted infection, is a leading cause of cancer, particularly cervical cancer. High-risk HPV types are strongly associated with almost all cases of cervical cancer (Graham, 2017; Wang and Xiao, 2022). After HR-HPV infection, viral DNA integrates into host cells, causing mutations and inhibiting tumor suppressor genes. This can upregulate oncogenes E6 and E7, leading to cell proliferation and potentially causing cervical cancer (Zheng and Li, 2015; Scarth et al., 2021). There is no standard treatment for HR-HPV.

Our study explored how QDF treats HR-HPV by combining UPLC-QTOF-MS analysis, network pharmacology, and cellular experiments. Analysis showed the presence of berberine, diammonium glycyrrhizinate, and phellodendrine in both positive and negative ionization modes, with indirubin and tubeimoside A only detected in the negative ionization mode. Berberine has broad antiviral effects by blocking reverse transcriptase, protecting the host with antioxidants, inhibiting virus-activated cell signaling, and balancing T cell subsets (Zhang and Shen, 2023). Gliquidone can reduce inflammation by inhibiting TNF-α signaling, lowering IL-6 and chemokine levels, and promoting apoptosis (Li and Li, 2019). β-sitosterol suppresses inflammatory factors, inhibits cell proliferation, induces apoptosis in tumor cells, promotes autophagy, and inhibits tubulin polymerization (Li et al., 2021). Quercetin fights viruses by blocking viral proteins and helicase activity, limiting infection. It also helps regulate interferons to reduce inflammation caused by the infection (Shi et al., 2022).

This study used network pharmacology to identify 141 active constituents of QDF and predict 254 target genes related to its effects on cervical HR-HPV. PPI analysis suggested TP53, JUN, AKT1, STAT3, TNF, and IL6 as potential treatment targets. AKT1, a key protein kinase, plays a vital role in regulating cellular processes (Ren et al., 2020). Quercetin targets AKT1 in the PI3K/AKT signaling pathway, which is linked to HPV16 infectionand the development of cervical lesions (Welcker et al., 2004). IL-6 has multiple functions as a cytokine, including anti-apoptotic effects by activating PI3K and inhibiting transforming growth factor (Zhu et al., 2017). HR-HPV infection is linked to higher IL-6 levels in cervicovaginal lavages and increased STAT3 expression in HPV positive individuals compared to HPV negative individuals (Yang, 2020; Hou et al., 2023).

QDF is linked to various biological processes and molecular categories. The IL-17 signaling pathway is crucial for immune response and inflammation. IL-17 binds to its receptor, activating the STAT3 pathway and leading to the release of proinflammatory cytokines through the MAPK and NF-κB pathways, amplifying the inflammatory response (Zhang, 2020). The TNF pathway is important for cell processes like growth, inflammation, and immunity. TNF-α is a key component, activating pathways essential for virus clearance and antitumor immunity. TNF-α expression is higher in HPV16 positive HSIL patients compared to HPV16 negative individuals (Shen et al., 2021). The PI3K/AKT pathway controls cell growth, differentiation, and death, and its abnormal activation is closely tied to tumor development and spread (Wang et al., 2007). Persistent HPV infection activates the PI3K/AKT pathway, leading to cervical lesion development. QDF and HR-HPV show strong binding to AKT1 in molecular docking analysis.

The experiments showed that QDF inhibited H8 cell proliferation in a dose-dependent manner. It induced apoptosis and cell cycle arrest, suppressed the PI3K/AKT pathway, and increased Caspase-3 and Bax expression while decreasing Bcl-2 expression. These results aligned with network pharmacology analysis.

In summary, this study used UPLC-QTOF-MS integrated network pharmacology to investigate how QDF treats HR-HPV. The study found that the treatment inhibits. HR-HPV multiplication by regulating multiple pathways related to cellular immunity. The study suggests that QDF may cause H8 cell apoptosis by blocking the PI3K/AKT pathway. It uses a comprehensive approach to study the effects of quercetin on HR-HPV, providing useful information for future research.

Our study has limitations, including the need to further explore how QDF activates PI3K/AKT and its potential effects on other molecular signaling pathways. The clinical relevance of these mechanisms is unknown, and further research is needed.

## Data Availability

The original contributions presented in the study are included in the article/supplementary material, further inquiries can be directed to the corresponding authors.

## References

[B1] Bowden S. J. Doulgeraki T. Bouras E. Markozannes G. Athanasiou A. Grout-Smith H. (2023). Risk factors for human papillomavirus infection, cervical intraepithelial neoplasia and cervical cancer: an umbrella review and follow-up Mendelian randomisation studies. BMC Med. 21 (1), 274. 10.1186/s12916-023-02965-w 37501128 PMC10375747

[B2] Chang F. X. Xuesi D. He L. Maomao C. Dian Q. S. Si Y. H. (2022). Cancer statistics in China and United States, 2022: profiles, trends, and determinants. Chin. Med. J. Engl. 135 (5), 584–590. 10.1097/cm9.0000000000002108 35143424 PMC8920425

[B3] Chen R. Y. Zhu L. P. Du L. Sun D. L. (2017). Evaluation of the efficacy of patulin in the treatment of persistent high-risk human papillomavirus infections. Maternal child health care China 32 (05), 910–911.

[B4] Fu X. N. Jin Y. (2023). Research progress on persistent infection of high risk human papillomavirus after cervical conization. Chin. Med. J. 58 (06), 608–612.

[B5] Graham S. V. (2017). The human papillomavirus replication cycle, and its links to cancer progression: a comprehensive review. Clin. Sci. (Lond). 131 (17), 2201–2221. 10.1042/CS20160786 28798073

[B6] Hill E. K. (2020). Updates in cervical cancer treatment. Clin. Obstet. Gynecol. 63 (1), 3–11. 10.1097/GRF.0000000000000507 31815773

[B7] Hou Y. Li Y. Li X. Li Q. (2023). The relationship between HPV infection and immune imbalance and abnormal cell proliferation in patients with cervical lesions. Hebei Med. 29 (08).

[B8] Li H. B. Wu K. X. Shi K. H. Tang L. Liang C. Y. (2021). Research progress on chemical constituents, pharmacological action and clinical application of Fritillaria ussuriensis. Chin. J. Traditional Chin. Med. 46 (17), 4314–4322. 10.19540/j.cnki.cjcmm.20210325.601 34581034

[B9] Li X. Li J. (2019). Research progress on pharmacological effects of active components of glycyrrhiza extract. Jiangsu Tradit. Chin. Med. 51 (5), 81–86.

[B10] Li X. M. Xiong Q. (2022). Progress in the treatment of high-risk human papillomavirus infections with interferon. J. Appl. Med. 38 (19), 2384–2389.

[B11] Liu J. M. (2021) Meta-analysis of external use of traditional Chinese medicine in the treatment of cervical HR-HPV infection and clinical efficacy of qingdu lotion. Guangzhou University of Chinese Medicine.

[B12] Lv S. S. (2022) Erjia dihuang decoction in the treatment of persistent cervical HPV infection with liver and kidney deficiency and damp-heat syndrome. Hunan University of Traditional Chinese Medicine.

[B13] Meenu J. Dhananjay Y. Urmila J. Vishal C. Arun K. Y. Bipin C. (2023). Epidemiology, molecular pathogenesis, immuno-Pathogenesis,Immune escape mechanisms and vaccine evaluation forHPV-associated carcinogenesis. Pathogens 12 (12), 1380. 10.3390/pathogens12121380 38133265 PMC10745624

[B14] Ojone I. Adeola O. (2023). Updates on HPV vaccination. Diagn. (Basel) 13 (2), 243. 10.3390/diagnostics13020243 PMC9857409 36673053

[B15] Ren B. C. Zang Y. F. Liu S. S. Cheng X. J. Yang X. Cui X. G. (2020). Curcumin alleviates oxidative stress and inhibits apoptosis in diabetic cardiomyopathy via Sirt1-Foxo1 and PI3K-Akt signalling pathways. J. Cell. Mol. Med. 24 (21), 12355–12367. 10.1111/jcmm.15725 32961025 PMC7687015

[B16] Scarth J. A. Patterson M. R. Morgan E. L. Macdonald A. (2021). The human papillomavirus oncoproteins: a review of the host pathways targeted on the road to transformation. J. Gen. Virol. 102 (3), 001540. 10.1099/jgv.0.001540 33427604 PMC8148304

[B17] Shen X. X. Dou B. B. Hua T. Qiu H. B. Qiu W. N. (2021). The expression of TNF-α, Il-12 and Il-10 in high-risk HPV 16 infected vaginal lavage fluid was correlated with the grade of cervical intraepithelial neoplasia. Chin. J. Hosp. Infect. 31 (9), 1419–1422.

[B18] Shi Z. H. Zeng J. L. Huang X. R. Peng Y. H. Su W. W. Wang Y. G. (2022). Research progress on antiviral activity of quercetin and its derivatives. Mod. Appl. Pharm. China 39 (18), 2412–2420.

[B19] Wang J. Karen K. Hauser J. Rossi M. R. Zhou Y. Conway A. (2007). Colon carcinoma cells harboring PIK3CA mutations display resistance to growth factor deprivation induced apoptosis. Mol. Cancer Ther. 6 (3), 1143–1150. 10.1158/1535-7163.MCT-06-0555 17363507

[B20] Wang P. Xiao Y. B. (2022). Advances in human papillomavirus infections and treatments. J. local Anat. Surg. 31 (12), 1107–1111.

[B21] Welcker M. Orian A. Jin J. P. Girm J. E. Harper J. W. Esiman R. N. (2004). The Fbw7 tumor suppress sorregulates glycogen synthase kinase3 phosphory lation-de-pendentc-Mycprotein degradation. Proc. Natl. Acad. Sci. U. S. A. 101 (24)), 9080–9090. 10.1073/pnas.0402770101 PMC428477 15150404

[B22] Yang G. L. (2020). Clinical significance of detection of IL-6, IL-17, IFN-γ and TGF-β in vaginal lavage fluid in patients with high-risk cervical HPV infection. Chin. J. Mod. Med. 22 (7).

[B23] Zhang D. (2020). Study on the relationship between the pathogenicity of HR-HPV infection and the local vaginas, Il-17, Il-23. Electron. J. Pract. Gynecol. Endocrinol. 7 (32), 2–3.

[B24] Zhang M. F. Shen Y. Q. (2023). Research progress on antiviral pharmacological action of berberine. anti-infective Pharm. 20 (03), 223–227.

[B25] Zhang T. Q. Li C. C. Zhang T. F. Wang M. Y. Cui S. N. Huo Q. (2021). To investigate the mechanism of astragaloside IV in alleviating PC12. Chin. J. Traditional Chin. Med. 46 (24), 6465–6473. 10.19540/j.cnki.cjcmm.20210902.702 34994139

[B26] Zheng C. J. Li G. R. (2015). Correlation between C-Fos and HR-HPV viral load in cervical lesions and cervical carcinoma. Chin. J. coal industry Med. 18 (1), 10–14.

[B27] Zhu R. R. Dai L. Dai W. (2017). The value of a combination of high-risk human papillomavirus and colposcopic cervical biopsy in the diagnosis of CIN and early invasive carcinoma. Hainan Med. 28 (3), 423–425.

